# Immunization with the M12-N, M12-C, and M12-N+C fusion peptides derived from the M12 protein elicited varying levels of protective immune responses against multiple serotypes of group A *Streptococcus*

**DOI:** 10.3389/fimmu.2025.1636591

**Published:** 2025-11-21

**Authors:** Xiaolan Zhang, Yue Ma, Rige Na, Wenli Hou, Emanuel Hanski, Qin Zhou

**Affiliations:** 1 School of Basic Medical Sciences, Harbin Medical University, Harbin, China; 2 Chengdu Ruiyi Biotechnology Co., Ltd., Chengdu, China; 3 Department of Microbiology and Molecular Genetics, The Institute for Medical Research, Israel-Canada (IMRIC), Faculty of Medicine, The Hebrew University of Jerusalem, Jerusalem, Israel

**Keywords:** M peptide, ketosteroid isomerase, vaccine, protective efficacy, group A streptococcus, infection

## Abstract

The M protein located on the surface of group A *Streptococcus* has been extensively researched as a promising vaccine candidate. However, issues such as potential cross-reactivity with human tissues and the impact of selection of M peptide sequences have raised concerns regarding the safety and efficacy of the M protein vaccine. In this study, we utilized a KSI (ketosteroid isomerase, 15.78 kDa) tag and conducted a comparative analysis of the N-terminal (M12-N, 28.14 kDa), C-terminal (M12-C, 30.24 kDa), and fusion form (M12-N+C, 29.19 kDa) derived from the M12 protein found in MGAS9429. Three vaccine candidates formulated with aluminum hydroxide adjuvant significantly increased specific antibody titers in serum following booster immunization. Furthermore, immunization with these vaccines improved the survival rates in mice challenged subcutaneously with MGAS9429 compared to control mice. The immune responses induced by our vaccine formulation were characterized by Th1 type responses marked by IFN-γ secretion rather than the Th2 type responses and a notable increase in effector memory T cells. Significantly, the vaccine candidate M12-C exhibited several advantages including shortened vaccination times, enhanced antibody levels, improved survival rates against non-vaccine serotype MGAS5005 challenge. Moreover, the M12-C antiserum demonstrated significant opsonization and killing effects on the non-vaccine strains of M1, M3, M6 and M18. This work identifies a promising fusion sequence of vaccine candidate when developing GAS vaccines based on M peptides to enhance immune responses and protective efficacy.

## 1 Introduction

*Streptococcus pyogenes*, also known as group A *Streptococcus* (GAS), is responsible for causing acute and chronic infectious diseases of significant global concern. It is estimated that there are 639,000 deaths annually due to rheumatic heart disease (RHD) and invasive GAS infections (1–3). Despite the substantial impact of these diseases, there is currently no commercially available vaccine for GAS (4–6). Due to the fact that the M protein is capable of inducing the generation of opsonophagocyte antibodies, M proteins including M1, 3, 6 and 14 had been extensively researched as vaccine candidates (7–10). However, the full-length M protein vaccine possesses a potential risk of RHD in humans (10). M peptide vaccines lacking cross-reactive fragments with human antigens are more promising compared to the M protein vaccines (11–17). The M peptide vaccine StreptInCor, composed of 55 amino acid residues from the C-terminal region of the M5 protein, demonstrates immunogenicity and the ability to opsonize M1, M5, M12, M22 and M87 GAS strains (11, 12). Another C-terminal vaccine known as J8, representing the minimal B cell epitope within p145 and conjugated to diphtheria toxoid (DT) or CRM197, has been shown to provide protection against GAS infection (13, 14). However, promising developments in the form of 26-valent and 30-valent N terminal vaccines are undergoing various stages of clinical trials (15–17). Approximately 59.28% of the detected strains in China belong to the M12 serotype, while 34.86% are classified as belonging to the M1 serotype (18). Therefore, our focus is on the predominant M12 serotype, and we aim to develop a monovalent or bivalent vaccine with a low molecular weight and minimal side effects. A previous study has revealed that the recombinant M1 protein provides 100% protection against the same serotype and possesses the ability to induce the production of bactericidal antibodies (19). Therefore, it is a viable option to select M peptides from M12 protein that do not have cross-reactivity with human tissue. However, the selection of M peptides may impact the effectiveness of vaccines. In this study, the hypervariable N-terminal sequence of the M12 protein was selected based on the first 50 amino acid residues of the mature protein following the removal of the signal peptide, while the conservative C-terminal sequence was selected based on a segment of 63 amino acid residues encompassing both the T-cell epitope and the B-cell epitope. The amino acid sequences were compared against human homologous protein sequences using blastp (https://blast.ncbi.nlm.nih.gov/Blast.cgi) respectively, and no homology was found. It has been tentatively concluded that these sequences may be exclusive to bacteria. Furthermore, KSI is a naturally occurring small protein that is found in prokaryotes. Its amino acid sequence exhibits minimal homology with mammalian proteins. In addition to its inherent immunogenicity, KSI can function as a carrier molecule, effectively amplifying the immune response to weak antigens. We selected it as the carrier for the M12 peptide to facilitate the proper folding of the antigenic peptide and enhance its expression level. Furthermore, GAS frequently leads to impetigo, therefore a mouse subcutaneous infection model was employed to assess the protective effect against infection in this study (20, 21). This study aims to evaluate the efficacy of N-terminal and C-terminal vaccines, as well as M12-N+C. The optimal sequence we have identified can also be combined with non-M protein sequences to construct novel GAS vaccines, thereby further improving the protective efficacy.

## 2 Materials and methods

### 2.1 Bacterial strains and culture condition

GAS was cultured in Todd-Hewitt broth (BD) supplemented with 0.2% yeast extract (THY) at 37 °C with 5% CO_2_. The bacteria were grown to the logarithmic phase in THY (OD_600_ = 0.6 to 0.8), then pelleted, washed and resuspended in phosphate buffered saline (PBS) at the desired concentration. The strain information utilized in this study is presented as follows: MGAS5005 (M1, ATCC: BAA-947), SF370 (M1, ATCC: 700294), MGAS10270 (M2, ATCC: BAA-1063), MGAS315 (M3, ATCC: BAA-595), MGAS10750 (M4, ATCC: BAA-1066), 19615 (M5, ATCC: 19615), MGAS10394 (M6, ATCC: BAA-946), MGAS9429 (M12, ATCC: BAA-1315), MGAS8232 (M18, ATCC: BAA-572), MGAS6180 (M28, ATCC: BAA-1064), NZ131 (M49, ATCC: BAA-1633), B514 (M50, ATCC: 12382), Alab49 (M53, ATCC: BAA-1323).

### 2.2 Gene synthesis, protein expression and purification

The full-length sequence of M12 protein (GenBank: ABF32911.1, 575 aa) was analyzed. The adjusted KSI sequence from *Comamonas* testosteroni (MGSSHHHHHHSSGLVPRGSHMHTPEHITAVVQRFVAALNAGDLDGIVALFADDATVEDPVGSEPRSGTAAIREFYANSLKLPLAVELTQEVRAVANEAAFAFTVSFEYQGRKTVVAPIDHFRFNGAGKVVSIRALFGEKNIHACQGS) and the sequences of M12-N (amino acids 42 to 91), M12-C (amino acids 376 to 438) and M12-N+C were synthesized together and cloned into the pET-28b (+) vectors (between NdeI and EcoRI) to produce the recombinant plasmids respectively. Subsequently, the recombinant plasmids were transformed into *E.coli* T7 Express in order to induce the expression of proteins upon induction with 1 mM IPTG at 37 °C, with shaking 200rpm for 4 hours. The bacterial precipitates were obtained through centrifugation, subsequently treated with sonication, and filtered. The recombinant proteins in supernatant were then purified using Ni resin affinity chromatography (HISTRAP HP, Cytiva). Protein was detected using a 12% SDS-PAGE and the concentration was determined with a BCA protein assay kit.

### 2.3 Vaccine formulation

The 50 μg of purified recombinant protein was diluted with PBS to a total volume of 50 μl. Subsequently, the protein solution was mixed with an equal-volume (50 μl) aluminum hydroxide adjuvant (ThermoFisher), generating three vaccine candidates: M12-N, M12-C, and M12-N+C respectively. The control mice were administered either 50 μg of recombinant KSI protein plus a 50 μl adjuvant (KSI-Alum) or a mixture of 50 μl PBS and an equal-volume adjuvant (PBS-Alum).

### 2.4 Mouse vaccination and challenge experiments

Female BALB/c mice (6 to 8 weeks old) were purchased from Liaoning Changsheng Biotechnology Co., Ltd. The mice received intramuscular immunization three times with 100 μl of protein plus adjuvant or PBS plus adjuvant on days 0, 14 and 28. The number of each group was as follows: M12-N (n=37), M12-C (n=37), M12-N+C (n=37), KSI-Alum (n=37), PBS-Alum (n=58). Body weight was measured every two weeks, and the sera were collected at specific time points. On day 42, the mice were challenged by subcutaneous injection with GAS at a dose of 2.0×10^8^ CFU per mouse. Subsequently, the survival rate of mice was monitored for a period of 15 days, and both skin lesion as well as cytokine levels were assessed to evaluate the efficacy of the vaccine. On day 56, mice were sacrificed by the cervical dislocation after being anesthetized with tribromoethanol (4 mg/10 g body weight) by intraperitoneal injection. All animal procedures were reviewed and approved by the Institutional Research Board of Harbin Medical University (Assurance Number: HMUIRB2024011).

### 2.5 Serum specific antibody titers

Blood samples were collected on days 0, 14, 28, 42 and 56 via tail bleeding and subsequently stored at -80°C after serum separation. Enzyme-linked immunosorbent assay (ELISA) was performed in a 96-well Costar plate coated with 100 μl recombinant protein M12-N, M12-C or M12-N+C (2 μg/ml in 50 mM sodium carbonate solution, pH 9.6) per well overnight at 4°C. Subsequently, the wells were blocked with 3% BSA at 37°C for 2 hours followed by washing the wells thrice with PBST containing 0.05% Tween to remove any excess protein. The gradient diluted antisera were then added into the wells for a duration of one hour. Then HRP-labeled goat anti-mouse IgG was added to each well for another hour prior to washing thrice and the TMB substrate solvent was added and incubated for ten minutes before adding 2M HCl to detect the absorbance values at 450nm. The final titer was determined as the A450nm value of the highest dilution of antisera, which exceeded three times the A450nm value of the 1:1000 dilution of pre-immunization sera.

### 2.6 Flow cytometric analysis

On days 19 and 33, mice were sacrificed by the cervical dislocation after being anesthetized with tribromoethanol (4mg/10g body weight) by intraperitoneal injection. Spleens were harvested from these immunized mice. A total of 10^6^ splenocytes were stimulated with leukocyte activation cocktail with BD GolgiPlug (BD Pharmingen, product No. 550583) for 5 hours at 37°C with 5% CO_2_ to block cytokine secretion. After stimulation, splenocytes were washed twice with PBS and surface-stained on ice for 20 minutes using fluorescently-labeled antibodies to CD3 (FITC), CD4 (PE), CD44 (PE-CY7), CD62L (Percp-cy5.5). Subsequently, the splenocytes were fixed and permeabilized with fixation/permeablization kit (BD Pharmingen, product No. 554714) at 4°C for 20 minutes, followed by washing. The intracellular staining was performed in the dark for 30 minutes using IL-4 (PE-CF594) and IFN-γ (APC) or an appropriate isotype control (All the above antibodies were from BioLegend). The dead cells were then stained with zombie NIR™ fixable viability kit (BioLegend), and samples were analyzed using a FACSVerse flow cytometer (BD Biosciences). Data were analyzed with the FlowJo software.

### 2.7 Opsonophagocytic killing assay

Briefly, 10 μl of GAS, washed and diluted to 2,000 CFU per well in opsonization buffer (composed of 10% [vol/vol] fetal bovine serum and 0.1% [wt/vol] gelatin in Hanks’ balanced salt solution containing Ca/Mg), was incubated at room temperature for 10 minutes with shaking at 700 rpm on a mini-orbital shaker alongside 20 μl of antisera that had been serially diluted in the opsonization buffer within a round-bottomed 96-well plate. Following this, 100 μl (≈1×10^6^) of differentiated human promyelocytic leukemia cells (HL-60) were added to each dilution of serum. This mixture was then incubated for one hour at 37°C with an atmosphere enriched with 5% CO_2_ while maintaining shaking at a speed of 700 rpm. Prior to the assay, HL-60 cells were cultured in IMDM medium supplemented with 15% fetal bovine serum (Gibco) until they reached the logarithmic growth phase (22). HL-60 cells underwent differentiation by incubation in a medium containing 0.8% dimethylformamide (DMF) at a controlled temperature of 37°C with an atmosphere comprising 5% CO_2_ for five days (see **Supplementary Figure S3**). Subsequently, the plates were placed on ice, and ten microliters from each well were spotted onto THY agar plates. These plates were incubated overnight at 37°C enriched with 5% CO_2_ and the colonies were counted the next day. The following acceptance criteria was adopted: non-specific killing of less than 35% and the maximum killing of at least 70% for test serum (23). The percent of killing was calculated by using the following formula: {[(CFU after 1 h of growth with preimmune serum) - (CFU after 1 h of growth with threefold diluted immune serum)] ÷ CFU after 1 h of growth with preimmune serum} × 100.

### 2.8 Cytokine quantification

The splenocyte samples collected from immunized mice on days 19 and 33, as well as the splenic homogenates were utilized for cytokine measurement. The samples or homogenate underwent centrifugation to obtain supernatant, from which the concentrations of IFN-γ and IL-4 were quantified using ELISA kits in accordance with the guidelines provided by the manufacturer (product No. E-EL-M0048 and E-EL-M0043, Elabscience Biotechnology Co., Ltd.).

### 2.9 Statistical analysis

The data were analyzed using Student’s *t*-test or paired *t*-test. The serum titers were evaluated by two-way ANOVA with Tukey’s multiple comparisons test. The log-rank test was utilized to analyze the survival rates of the mice. All figures were plotted using GraphPad Prism version 8. P values less than 0.05 were considered as statistically significant.

## 3 Results

### 3.1 The antigen design of vaccine candidates

**Figure 1:**
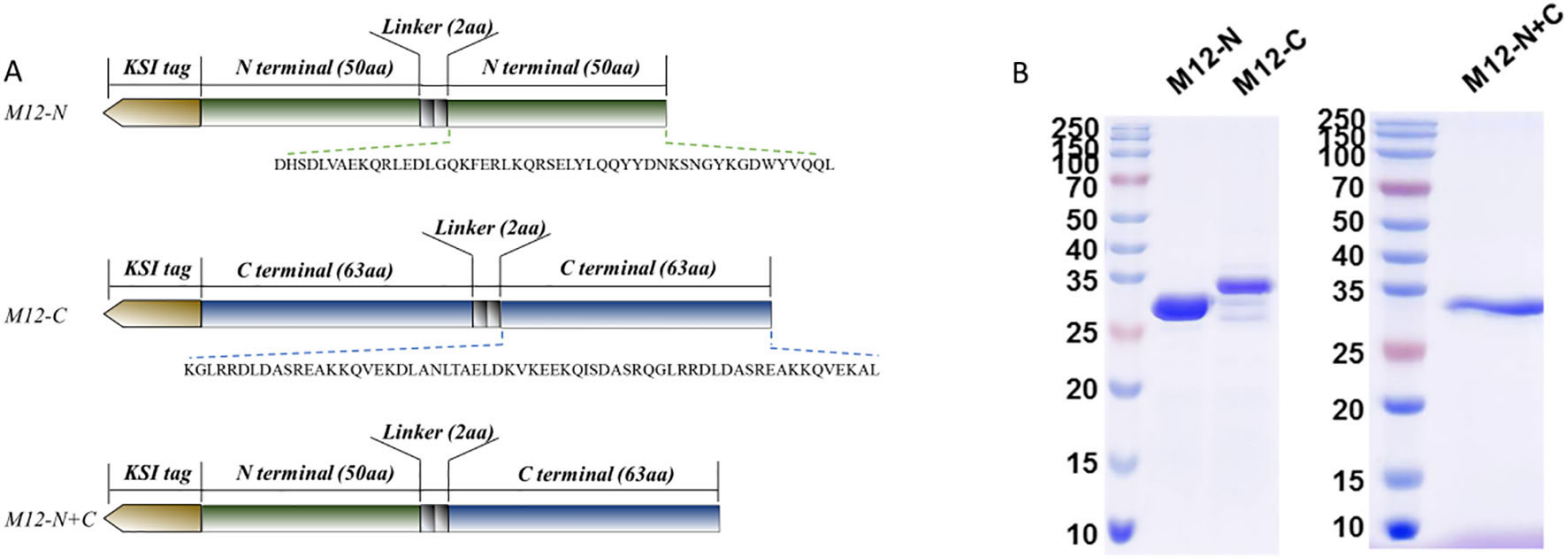
The antigen constructs and proteins detected by SDS-PAGE. **(A)** The antigen constructs of the vaccine candidates involved repeating and connecting the M-peptide sequence with a GS linker. **(B)** The recombinant plasmids were transformed into *E.coli* T7 Express and recombinant proteins were expressed and detected.

The complete sequence of the M12 protein (GenBank: ABF32911.1) was aligned with multiple M proteins from serotypes *emm* 1, 3, 4, 5, 6, 18, and 49 to 59. Both the type-specific N-terminal sequence (amino acids 42 to 91) and relatively conserved C-terminal sequence (amino acids 376 to 438) of the M12 protein were selected as antigen sequences. Subsequently, a KSI tag was added to the front of each sequence and a GS linker was inserted in the middle of the antigenic peptide. This resulted in three combinations as depicted in **Figure 1A** and they were named M12-N, M12-C, and M12-N+C respectively. The recombinant proteins were verified by electrophoresis (**Figure 1B**).

### 3.2 Mice exhibited weight gain after immunization with three vaccine candidates that induced high levels of specific antibodies in serum

**Figure 2:**
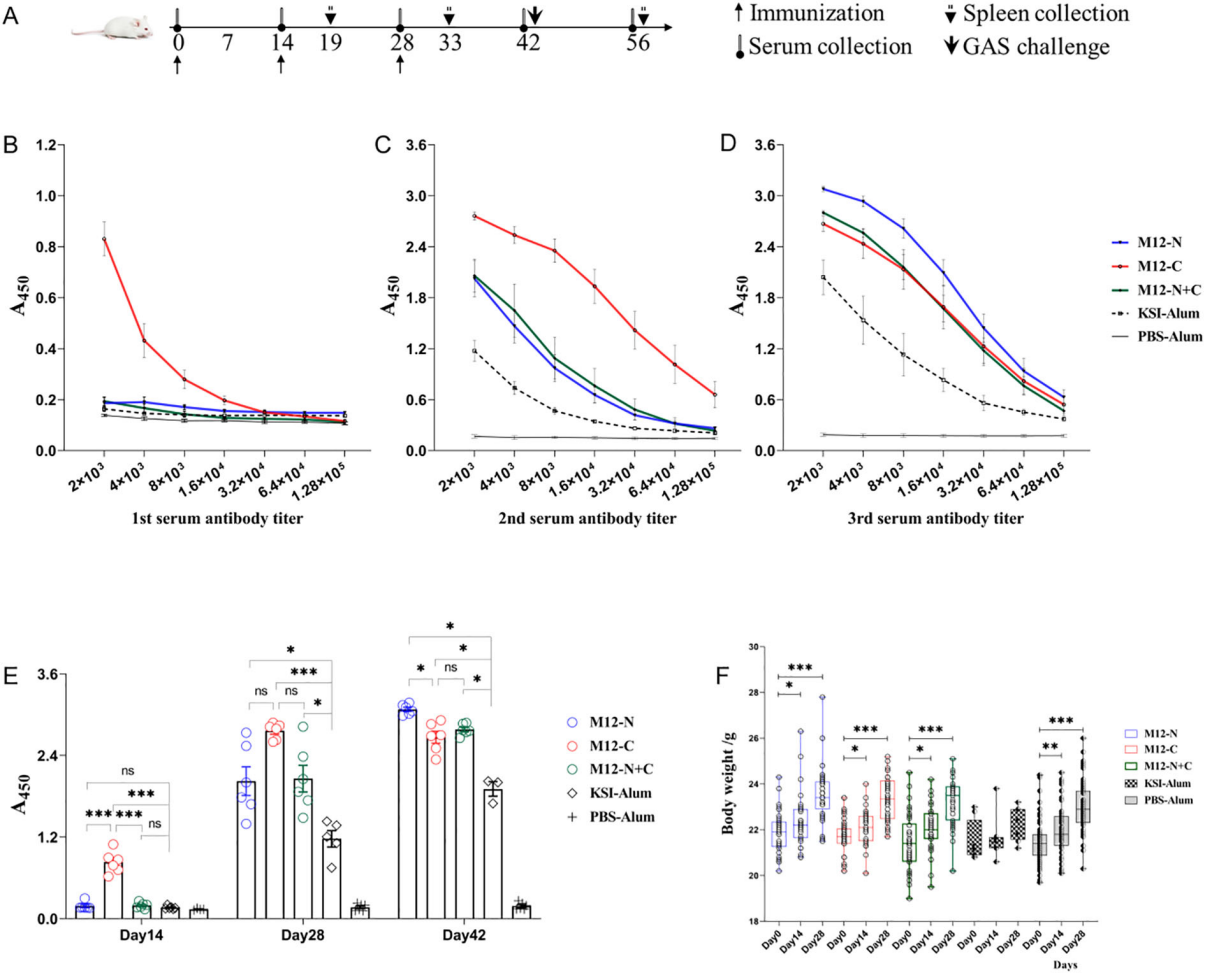
The serum antibody levels and body weights of immunized mice. **(A)** Schematic diagram illustrating the immunization process, sample collection, and challenge schedule. **(B-D)** Serum antibody levels determined by ELISA two weeks after each immunization. Data are represented as the mean ± SEM, n=6 for each group. **(E)** Statistical graph depicting the serum antibody levels. Data are represented as the mean ± SEM, n=6 for each group. **(F)** Changes in body weight throughout the vaccination process. Data are represented as the mean ± SD, M12-N (n=37), M12-C (n=37), M12-N+C (n=37), KSI-Alum (n=8), PBS-Alum (n=58). *P < 0.05, **P < 0.01, ***P < 0.001.

Mice were vaccinated or treated according to the immune procedure shown in **Figure 2A**. ELISA was utilized to detect the specific antibody levels directed against antigens. The vaccine candidate M12-C significantly elevated the antigen-specific serum antibodies after the first immunization, yielding an endpoint titer of 1:128000 after the second immunization (**Figures 2B, C**). In contrast, both M12-N and M12-N+C induced a titer of 1:32000 after the second immunization (**Figure 2C**). Subsequent to the third immunization, M12-N exhibited an endpoint titer of 1:128000, while M12-N+C had a titer of 1:64000 (**Figure 2D**). In comparison with KSI-Alum and PBS-Alum, all three vaccine candidates elicited higher levels of specific antibodies (**Figure 2E**). Meanwhile, the average weight of the mice gradually increased, and there were no fatalities during the immunization process (**Figure 2F**). These findings provide further evidence of the safety of the vaccine formulations. It is important to note that M12-C offers advantages in terms of reducing the number of required vaccinations and enhancing antibody levels.

### 3.3 Immunization with the three vaccine candidates protected mice against MGAS9429 infection

**Figure 3:**
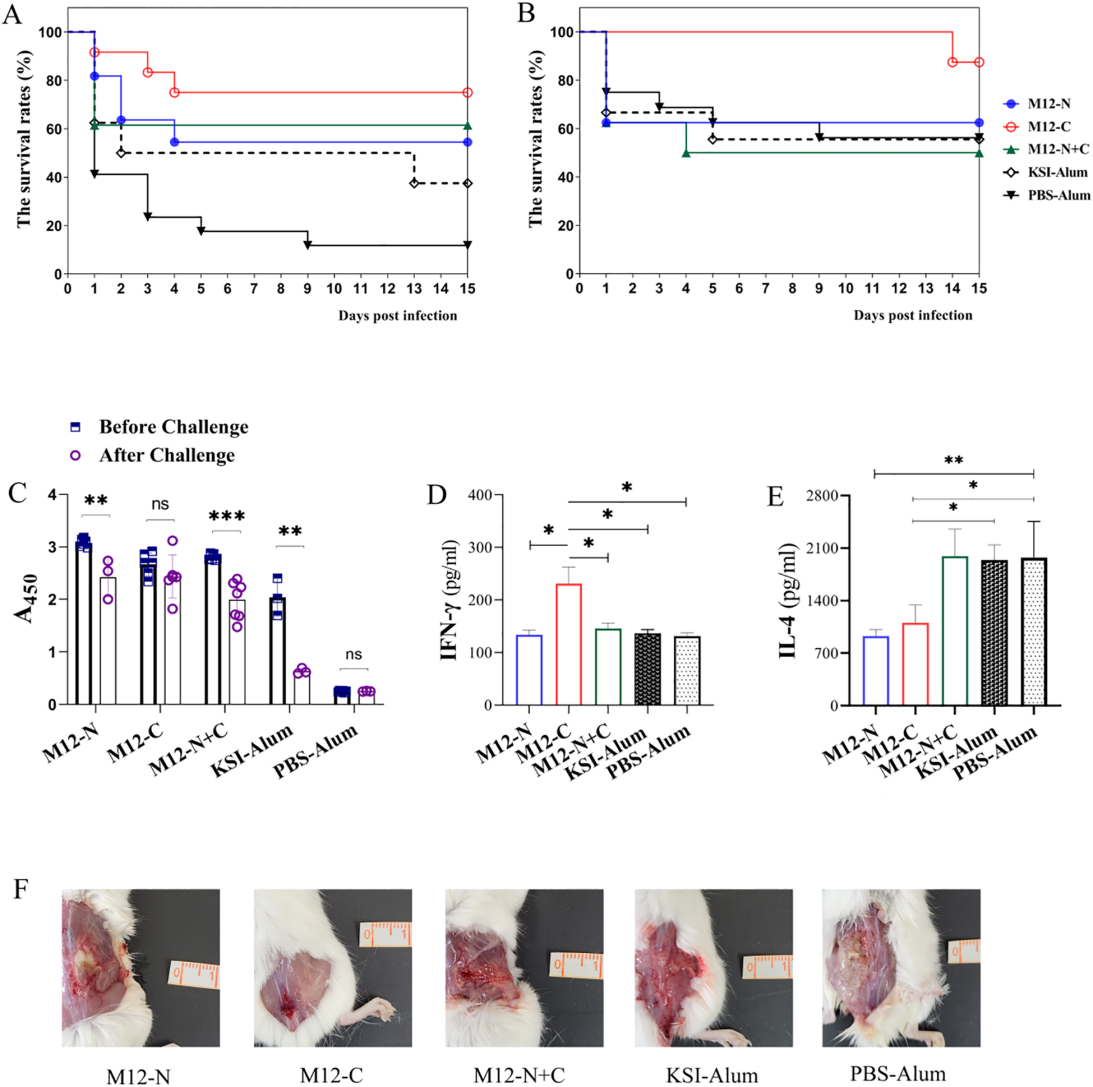
Efficacy evaluation against GAS challenge in mice. The efficacy of the immunization was evaluated against GAS challenge in mice, with the survival rates of immunized mice challenged by **(A)** MGAS9429, n=10 for each group and **(B)** MGAS5005, n=8 for each group being assessed. The mice received a subcutaneous inoculation via the dorsal surface with 2.0×10^8^ CFU and the survival rates were analyzed using the log-rank test. **(C)** Serum antibody titers of 1:2000 were compared before and after MGAS9429 challenge to assess immune response. ELISA plates were coated with respective antigens. Data are represented as the mean ± SD, n=6 for each group besides KSI-Alum (n=3). Statistical analysis was performed using a paired *t*-test. The levels of IFN-γ **(D)** and IL-4 **(E)** in the splenic homogenates of mice infected with 2.0 × 10^8^ CFU MGAS9429 were measured. Data are represented as the mean ± SD, n=4 for each group. **(F)** Representative pictures of skin lesion of immunized mice post challenge. *P < 0.05, **P < 0.01, ***P < 0.001.

To assess the efficacy of vaccination in providing protection against GAS infection, mice were subcutaneously challenged with MGAS9429 or MGAS5005. As shown in **Figure 3A**, at day 15 post-MGAS9429 challenge, it was observed that 75% of M12-C immunized mice survived, while only 11.76% of PBS-Alum immunized mice (Log-rank test: M12-C vs. PBS-Alum *P* = 0.0005 and vs. KSI-Alum *P* = 0.0469), 54.54% of M12-N immunized mice (vs. PBS-Alum *P* = 0.0230 and vs. KSI-Alum *P* = 0.3502) and 61.53% of M12-N+C immunized mice survived (vs. PBS-Alum *P* = 0.0091 and vs. KSI-Alum *P* = 0.2728) respectively. All mice immunized with PBS-Alum became moribund whereas a 33.3% survival rate was observed for KSI-Alum immunized mice (vs. PBS-Alum *P* = 0.2122, not significant). The combined immunization with KSI tag and aluminum adjuvant resulted in an elevation of the corresponding antibody levels (**Figure 2E**). This unexpected immune protective effect may be associated with the elevated antibody levels in mice induced by KSI immunization as a heterologous protein. Nevertheless, this effect might not be sufficient to confer protective immunity against infection in mice. Our experiments in rabbits also demonstrated that KSI immunization resulted in increased serum antibody levels (see **Supplementary Table S1**). These results indicate that the protective effects against MGAS9429 challenge are partly attributed to KSI vaccination but mostly led by the three vaccine antigens. Furthermore, it was observed that immunization with M12-C provided more robust protection against the non-vaccine serotype MGAS5005, and even achieved a 87.5% survival rate (**Figure 3B**). In contrast, both M12-N and M12-N+C failed to confer protection against MGAS5005. In addition, on the 7th day post infection, the number of bacterial colonies in skin inoculation site, spleen, and kidneys of the surviving mice was assessed. The results indicated that the vaccinated groups, particularly the M12-C group, exhibited a statistically significant reduction in colony counts compared to the control group (see **Supplementary Figure S1**). Our studies suggest that M12-C is superior to its counterparts in providing protection against GAS infections.

Moreover, mice immunized with M12-C maintained a higher level of antibodies compared to those immunized with M12-N or M12-N+C after the challenge (**Figure 3C**). The antibody level in M12-C immunized mice did not decrease significantly and was able to sustain a high level after the MGAS9429 challenge. In contrast, the antibody levels in KSI-Alum immunized mice decreased notably, correlating with a relatively low survival rate among the mice. Fifteen days after challenge with MGAS9429, the splenic homogenate of mice immunized with M12-C exhibited a higher level of IFN-γ and a lower level of IL-4 compared to control mice (**Figures 3D, E**). The surviving M12-C immunized mice showed a robust Th1-type immunity following the MGAS9429 challenge. In addition, it can be seen from the skin lesion photos of mice that the inflammatory reaction of mice immunized with M12-C was mildest, while mice immunized with PBS-Alum was severest (**Figure 3F**). The abscess was deeper and larger in the control mice. These findings indicate that immunization with M12-C may offer prolonged protection post infection.

### 3.4 The vaccine candidates primarily induced Th1-type immunity, and increased the proportion of effector memory T cell

**Figure 4:**
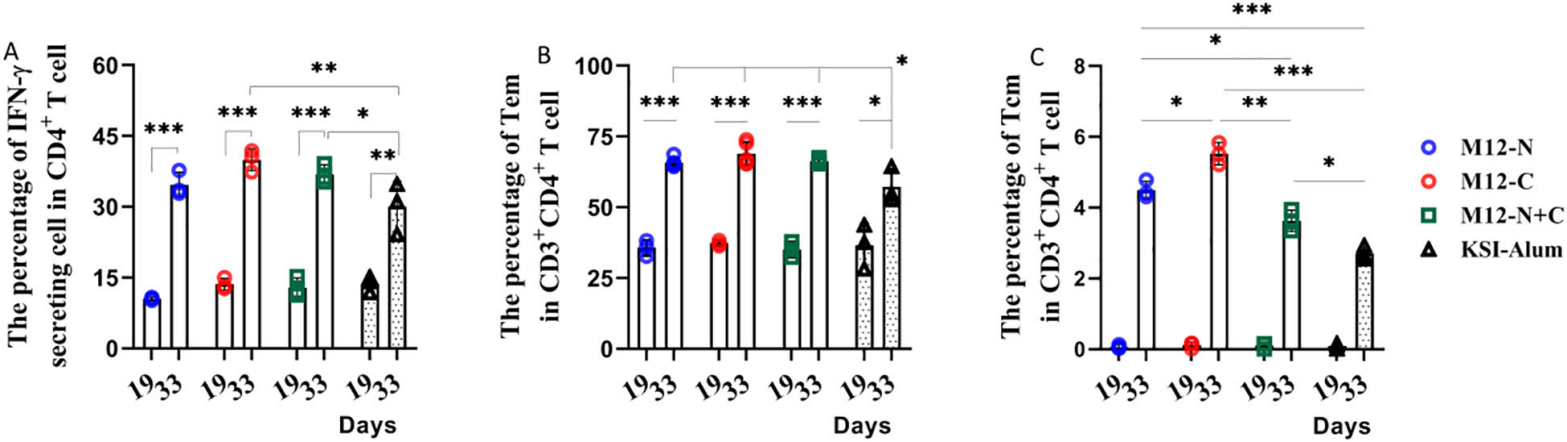
Immunization elicited a higher frequency of CD4^+^ IFN-γ producing T cells and CD4^+^ Tem cells predominate following the third immunization. **(A)** The percentage of IFN-γ secreting cells, **(B)** Tem and **(C)** Tcm in CD3^+^CD4^+^ T cells was determined. Data are represented as the mean ± SD, n=3 for each group, showing an increase with statistical significance indicated by *P < 0.05, **P < 0.01, ***P < 0.001.

To assess the cellular immunity induced by the vaccine candidates, splenocytes and splenic homogenates from immunized mice were harvested. The levels of IFN-γ and IL-4, which represent Th1 and Th2 type immunity respectively, were measured. The proportion of CD4^+^ IFN-γ producing T cells significantly increased after the third immunization compared to those after the second immunization (**Figure 4A**). This indicates that the vaccination can enhance the differentiation of CD4^+^ T cells into Th1 effector cells and regulate the immune response by increasing IFN-γ generation. However, the IL-4 levels remained low both prior to and following immunizations (see **Supplementary Figure S2**). We proposed that the enhanced Th1 response observed was not a divergence from an effective pathway, but rather a crucial component of a multifaceted defense system that equipped macrophages and neutrophils as a rapid first line of bacterial clearance. IFN-γ itself can further optimize the humoral response by promoting B-cell class-switching to potent opsonic IgG subclasses (24, 25). In conclusion, this study observed Th1 responses, particularly increased IFN-γ production, playing a significant role in protective immunity against GAS infection.

Moreover, this study investigated the alterations in memory T cells of immunized mice. These findings were presented in **Supplementary Figure S2**, which illustrated a significant increase in the proportion of effector memory T cells (Tem) from just over 30% to more than 60% after the third immunization. This demonstrates that the third immunization notably heightened the proportion of Tem and central memory T cells, Tcm (**Figures 4B, C**). It is likely that the vaccine candidates prolong the duration of immune protection by enhancing the quantity of memory T cells.

### 3.5 The antiserum showed varying degrees of opsonization-mediated phagocytic killing effects against non-vaccine serotypes

**Figure 5:**
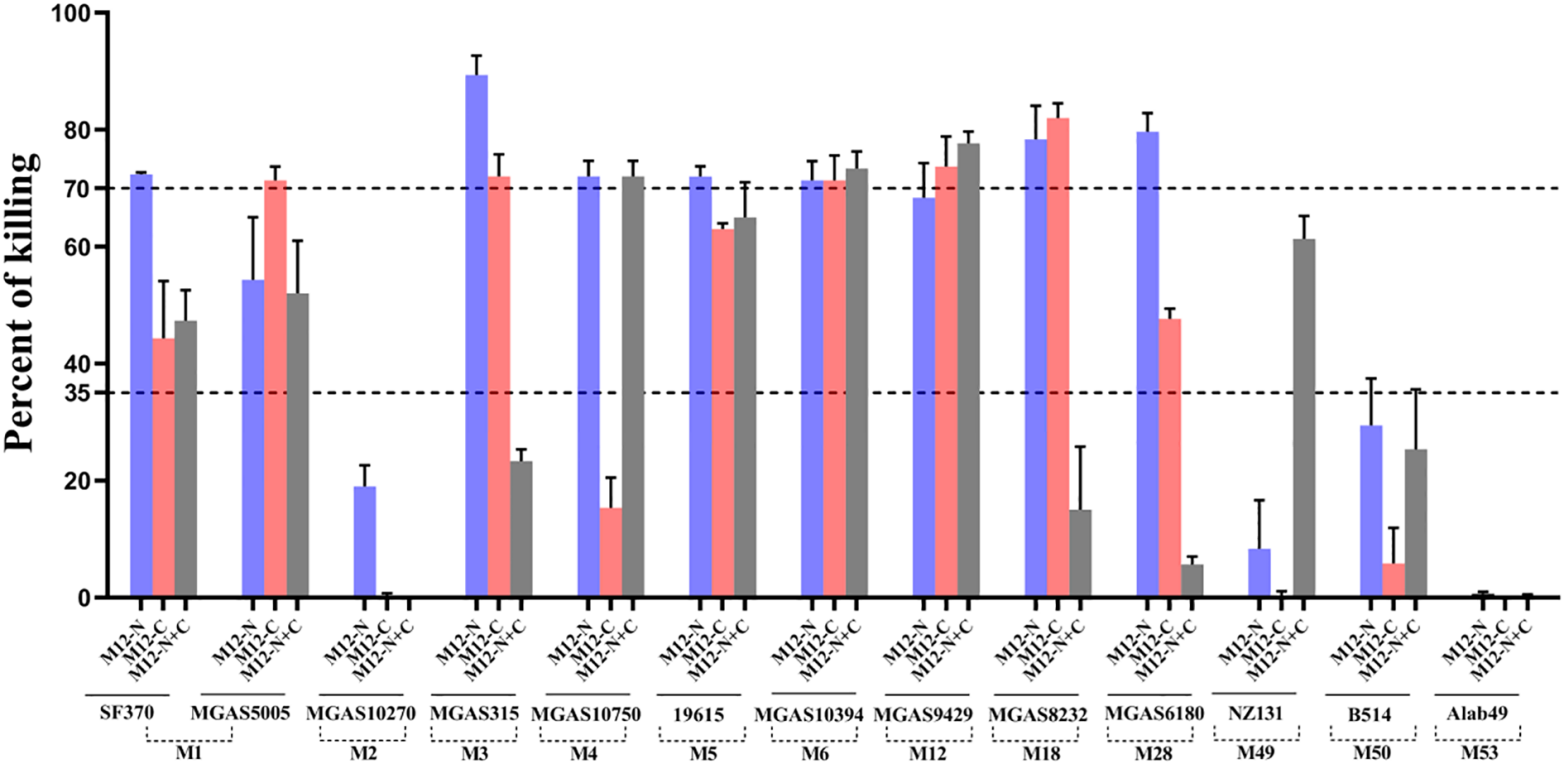
The opsonophagocytic killing assay of HL-60 cells using antisera against GAS. The OPK assay using GAS with antiserum (M12-N, M12-C, M12-N+C), specifically targeting the M peptides. The X-axis represented the groups in which the 3-fold diluted antiserum mediated the effects of multiple GAS serotypes, while the Y-axis denoted the percentage of killing. The two dashed lines corresponded to killing percentages of 35% and 70%, respectively. Each bar represented the mean value of three replicate measurements.

To investigate whether immunization with the M12-N, M12-C and M12-N+C could provide broad spectrum protection against non-vaccine serotypes, multiple GAS serotypes (M1, M2, M3, M4, M6, M12, M18, M28, M49, M50, M53) were chosen for opsonophagocytic killing (OPK) assay, as they are epidemiologically relevant and belong to different emm clusters. The antisera from M12-N or M12-C demonstrated varied levels of killing efficiency compared to the preimmune sera. The M12-N antiserum was capable of opsonizing the strains of M1, M3, M4, M5, M6, M18, M28, but no notable effect was observed on M2, M49, M50 or M53 (**Figure 5**). The strains of M1, M3, M6, M12 and M18 were effectively opsonized and killed by the M12-C antiserum. The protective efficacy of the M12-N+C antiserum was relatively less pronounced compared to the M12-N antiserum administered individually, however, it demonstrated partial protection against the NZ131. It observed that the antiserum exhibited varying degrees of opsonophagocytic killing effects on M1, M3, M5, M18 and M28 as dose-dependent (data not shown). These results indicated that the differences in protection may be attributed to distinct antibody-mediated antigen recognition and binding to the GAS surface, which in turn influenced the subsequent initiation of phagocytosis. These results demonstrated that the bactericidal activities of the M12-N, M12-C and M12-N+C antisera were not limited to the M12 serotype.

## 4 Discussion

Previous researchers have confirmed the safety and efficacy of multiple M peptide vaccines (11–17). The aim of this study was to develop an effective vaccine candidate that demonstrates strong protective efficacy against the predominant M12 serotype in China. They were derived from the N and C terminals of M12 protein. To increase the solubility and stability, a KSI sequence and a GS linker were added to the recombinant M peptide. KSI can function as a protein carrier, thereby augmenting the host immune response towards the target protein. The enhancements included a decrease in the molecular weight of the antigen, as well as improvements in antigen stability and immunogenicity. All three vaccine candidates elicited high levels of antigen-specific serum antibodies, particularly M12-C, which required only one booster to achieve the optimal titer. The level of serum antibody can be maintained even after GAS infection. We evaluated the efficacy of the vaccine candidates against infection and observed that all three were able to protect mice from MGAS9429 infection when compared with KSI-Alum or PBS-Alum. Notably, M12-C immunization significantly improved the survival rate and also protected against non-vaccine serotype MGAS5005 infection. These results demonstrate that the C-terminal of M12 protein, M12-C, is more effective in activating long-lasting immune responses and providing better protection against infection than either M12-N or M12-N+C. The original design intention of the fusion vaccine was to enable antibodies to provide protection against both the N and C termini, however, experimental results indicated otherwise. There may be mutual masking occurring within the fusion peptides, which hinders certain key epitopes from being fully accessible to the immune system. In contrast, epitopes derived from single sequence proteins are relatively straightforward and tend to be more readily recognized and bound by immune cells.

Compared to the recombinant full-length M protein vaccine, the M12 peptide vaccine eliminates cross-reactive antigenic sequences, thereby reducing the potential risk of rheumatic heart disease. In comparison with the M peptide vaccine J8-DT/Alum (MJ8Vax), the M12 peptide vaccine demonstrates a significantly enhanced protective efficacy against highly virulent *cov*R/S mutant strains. Furthermore, when compared to 26-valent and 30-valent N terminal vaccines, the M12 peptide vaccine markedly decreases the molecular weight of the antigen and achieves a protective coverage rate of 53.8% against non-vaccine serotypes.

The presence of Th1 type cytokine IFN-γ suggests a potential role for Th1 responses in protecting against infection following vaccination (26–28). However, the study did not show an enhanced Th2 type immune response represented by IL-4 secretion. This observation highlights the importance of Th1 responses, particularly IFN-γ, in conferring protective immunity against GAS infections. In addition, the immune memory is capable of recalling each specific stimulus and initiating a secondary response that is both faster and stronger than the primary response. It has been observed that CD4^+^ T cell memory can persist for at least 5 years (29, 30). Following the third immunization, there was a significant increase in the proportion of effector memory T cells compared to the second immunization. The booster immunization served to stimulate long-lasting immunity. However, even in the absence of antigens, KSI plus aluminum adjuvant can facilitate Th1 cell activation and IFN-γ secretion to some extent in this study. This may involve multiple immune regulatory mechanisms, particularly nonspecific activation of immune cells. Nevertheless, this activation effect failed to result in an improvement in protection in response to infection.

The distribution of prevalent GAS strains in China from 1990 to 2020 was investigated. A total of 8,820 GAS strains could be classified into 60 *emm* types (18). The most common GAS prevalent strains in China are *emm* 12 (59.28%) and *emm*1 (34.86%), accounting for 94.14% of all prevalent strains in total. Apart from *emm* 12 and *emm* 1, the top ten prevalent GAS strains also include *emm* 4, 22, 3, 75, 6, 18, 89 and 110 (18). To investigate whether immunization could provide broad spectrum protection against non-vaccine serotypes, we did a series of OPK assay, as those GAS serotypes are epidemiologically relevant and belong to different *emm* clusters. It observed that the antiserum exhibited varying degrees of opsonophagocytic killing effects on non-vaccine serotypes. These findings indicate that the antiserum not only provides protection against the M12 serotype but also exhibits protective effects against non-vaccine serotypes. This characteristic may represent a significant advantage in the development potential of the M12 peptide vaccine.

In conclusion, the M peptide vaccine derived from the C-terminal of the M12 protein exhibits more advantages compared to that derived from the N-terminal. Specifically, M12-C stands out as a promising candidate for a vaccine against the prevalent M12 serotype in China. Future research will focus on incorporating non-M protein epitopes, with the aim of developing highly immunogenic and effective vaccines that work synergistically to prevent multiple GAS serotypes.

## Data Availability

The original contributions presented in the study are included in the article/**Supplementary Material**. Further inquiries can be directed to the corresponding author/s.
