# Supplementary material for: Immunization with the M12-N, M12-C, and M12-N+C fusion peptides derived from the M12 protein elicited varying levels of protective immune responses against multiple serotypes of group A Streptococcus
Source: Front Immunol. 2025 Nov 21;16:1636591. doi: 10.3389/fimmu.2025.1636591 (PMC12678301; doi:10.3389/fimmu.2025.1636591)
Supplement: Supplementary file 1 [file DataSheet1.pdf]

Supplementary Materials for

Immunization with the M12-N, M12-C, and M12-N+C fusion peptides derived from the M12 protein elicited varying levels of protective immune responses against multiple serotypes of group A *Streptococcus*

Table S1 ELISA assay for serum antibody detection in rabbits following three vaccinations.

Dilution	Rabbit-1		Rabbit-2		Rabbit-3		Rabbit-4	
	M12-N	KSI-Alum	M12-N	KSI-Alum	M12-C	KSI-Alum	M12-C	KSI-Alum
1:2000	2.81	2.14	2.60	1.09	4.06	1.06	3.96	1.14
1:4000	1.90	1.45	1.65	0.57	3.34	0.63	3.28	0.73
1:8000	1.31	0.96	1.12	0.41	2.93	0.45	2.88	0.49
1:16000	0.85	0.65	0.70	0.31	2.41	0.32	2.41	0.36
1:32000	0.55	0.46	0.42	0.23	1.71	0.27	1.78	0.29
1:64000	0.46	0.33	0.31	0.23	1.27	0.23	1.30	0.26
1:128000	0.28	0.25	0.22	0.19	0.84	0.16	0.89	0.26

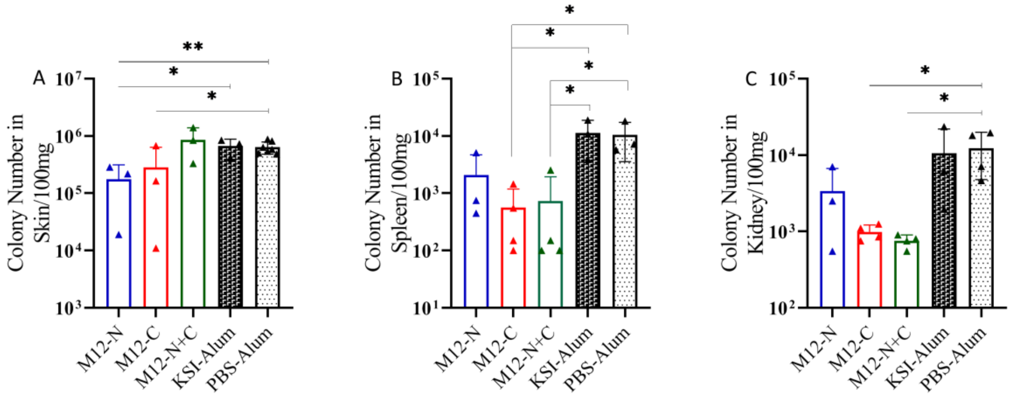

Figure S1 Bacterial CFU numbers in the skin, spleen and kidneys seven days following subcutaneous challenge with MGAS9429.

The organ homogenates were serially diluted in PBS and plated on THY agar plates for the enumeration of bacterial CFUs from (A) skin, (B) spleen, and (C) kidneys. All the data are represented as the mean ± SD, *P<0.05, **P<0.01.

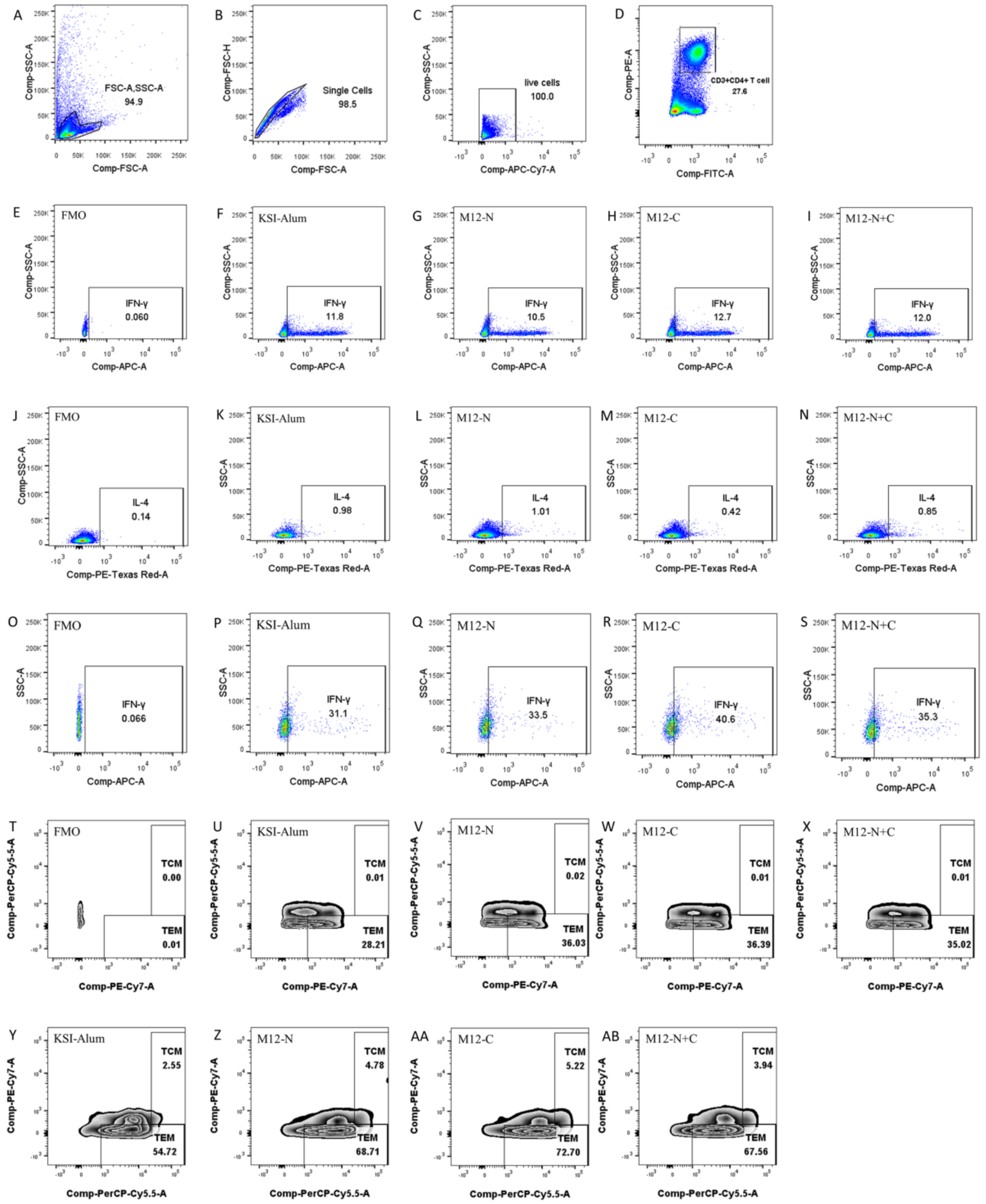

Figure S2. Immunization elicited a higher frequency of CD4$^+$ IFN-γ producing T cells and CD4$^+$ Tem cells predominate following the third immunization.

Specifically, splenocytes from each group at Day 19 (E-N, T-X) and Day 33 (O-S, Y-AB) were stained for CD3 (FITC), CD4 (PE), IFN-γ (APC), CD44 (PE-CY7), CD62L (Percp-cy5.5), IFN-γ (APC) and IL-4 (PE-CF594). Data are represented as the mean ± SD, n=3 for each group. Within the CD4+ T cell population, Tem and Tcm were identified as CD44$^+$CD62L$^-$ and CD44^{high}CD62L$^+$, respectively.

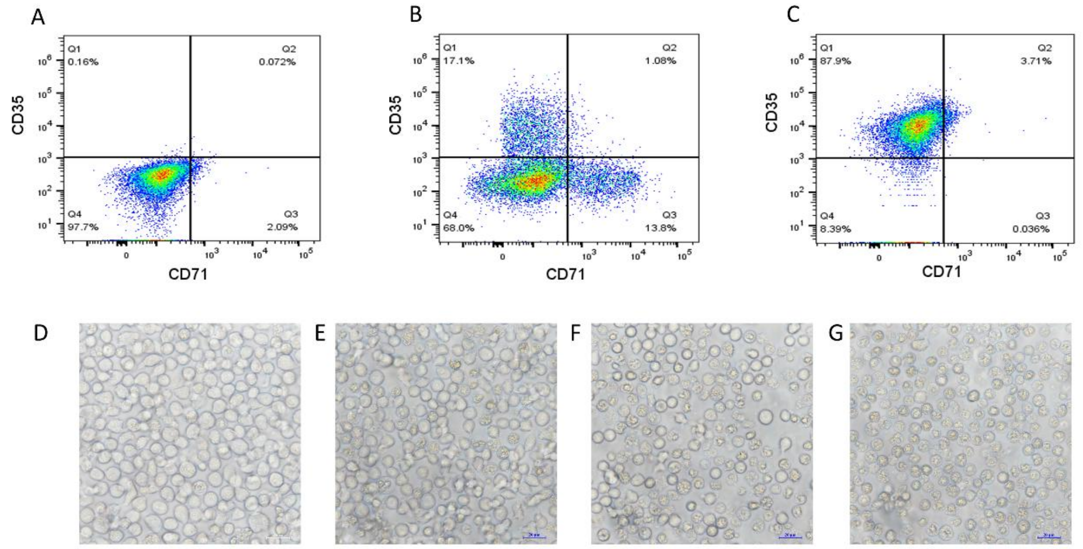

Figure S3. Undifferentiated HL-60 phenotype in comparison to its differentiated counterpart.

(A) The viability staining control. (B) The undifferentiated HL-60 cells exhibited a percentage of 17.1% for $CD35^{+}$ and 13.8% for $CD71^{+}$. (C) The differentiated HL-60 cells exhibited 87.9% positivity for CD35 and 0.036% for CD71. (D) The undifferentiated HL-60 cells exhibited a range of sizes, and their nuclei were not segmented into distinct lobes. (E) Following a two-day induction, HL-60 cells exhibited an increased presence of lobulated nuclei and organelles. (F) Following a four-day induction, the maturation level of the cells continued to increase. (G) Following a five-day induction, the number of lysosomes and organelles within the cells increased markedly, and the nuclei exhibited a more pronounced lobulated morphology.
